# Blind Exchange With Mini-Pinning Technique Using the Tron Stent Retriever for Middle Cerebral Artery M2 Occlusion Thrombectomy in Acute Ischemic Stroke

**DOI:** 10.3389/fneur.2021.667835

**Published:** 2021-05-19

**Authors:** Takeshi Yoshimoto, Kanta Tanaka, Junpei Koge, Masayuki Shiozawa, Hiroshi Yamagami, Manabu Inoue, Naruhiko Kamogawa, Tetsu Satow, Hiroharu Kataoka, Kazunori Toyoda, Masafumi Ihara, Masatoshi Koga

**Affiliations:** ^1^Department of Neurology, National Cerebral and Cardiovascular Center, Suita, Japan; ^2^Division of Stroke Care Unit, National Cerebral and Cardiovascular Center, Suita, Japan; ^3^Department of Cerebrovascular Medicine, National Cerebral and Cardiovascular Center, Suita, Japan; ^4^Department of Stroke Neurology, National Hospital Organization Osaka National Hospital, Osaka, Japan; ^5^Department of Neurosurgery, National Cerebral and Cardiovascular Center, Suita, Japan

**Keywords:** BEMP technique, M2 occlusion, Tron stent retriever, thrombectomy, acute ischemic stroke

## Abstract

**Introduction:** The usefulness of the blind exchange with mini-pinning (BEMP) technique has recently been reported for mechanical thrombectomy in patients with stroke owing to medium vessel occlusion (MeVO). The Tron stent retriever can be delivered and deployed through a 0.0165-inch microcatheter. This retriever has potential as an effective and safe treatment for acute ischemic stroke (AIS) due to occlusion of the M2 segment of the middle cerebral artery (MCA). Here, we report the outcomes of the BEMP technique using Tron stent retrievers for M2 occlusion thrombectomy.

**Methods:** Consecutive patients with AIS owing to M2 occlusion who underwent the BEMP technique using 2 × 15-mm or 4 × 20-mm Tron stent retrievers were included. The technique involves deploying a Tron stent retriever through a 0.0165-inch microcatheter, followed by microcatheter removal and blind navigation of a 3MAX or 4MAX aspiration catheter over the bare Tron delivery wire until the aspiration catheter reaches the clot. A Tron stent retriever is inserted into the aspiration catheter like a cork and subsequently pulled as a unit. We assessed procedural outcomes [first-pass expanded thrombolysis in cerebral infarction (eTICI) score 2c/3 and 2b/2c/3], safety outcomes [symptomatic intracranial hemorrhage (sICH)], and clinical outcomes (good outcome rate defined as modified Rankin Scale score 0–2 at 90 days and mortality at 90 days).

**Results:** Eighteen M2 vessels were treated in 15 patients (six female, median age: 80 years, and median National Institutes of Health Stroke Scale score: 18). The BEMP technique was performed successfully in all cases. Whether to use a 3MAX or 4MAX catheter was determined by considering one of the following target vessels: dominant, non-dominant, or co-dominant M2 (3MAX, *n* = 9; 4MAX, *n* = 9). The first-pass eTICI 2c/3 and 2b/2c/3 rates were 47 (7/15) and 60% (9/15), respectively; sICH was not observed. Seven patients (47%) achieved good outcomes, and one patient (7%) died within 90 days.

**Conclusions:** The Tron stent retriever was safely and effectively used in the BEMP technique for acute MCA M2 occlusion and can be combined with a 0.0165-inch microcatheter, which may be useful for treating MeVO, in general.

## Introduction

The usefulness of the blind exchange with mini-pinning (BEMP) technique has recently been reported for mechanical thrombectomy in patients with acute ischemic stroke (AIS) owing to medium vessel occlusion (MeVO), i.e., occlusions of the M2/M3 middle cerebral artery (MCA), A2/A3 anterior cerebral artery, and P2/P3 posterior cerebral artery segments (1, 2).

The BEMP technique, which is the blind catheter exchange technique and originally devised using a Trevo 3 × 20-mm retriever (Stryker, Fremont, CA, USA) delivery wire to advance the thromboaspiration catheter and the pinning technique with small devices (“mini-pinning”) for thrombectomy of intracranial distal occlusions, has been reported to be helpful and safe for optimizing procedural performance when treating MeVO (1, 2). Although original mini-pinning was required to pull the stent retriever slightly and lock the stent retriever and aspiration catheter (1), performing this operation for MCA distal M2 occlusion is challenging. The Tron stent retriever is a new stent retriever for AIS owing to not only large intracranial vessel occlusion but also MeVO (3). As the most significant feature, the Tron stent retriever can be deployed *via* a relatively small 0.0165-inch (0.42-mm) microcatheter, similar to the Aperio (Acandis, Pforzheim, Germany) (2 × 16 mm and 3.5 × 28 mm) (4, 5) or Catch View mini (Catch View; Balt, Montmorency, France) (3.5 × 20 mm), (6) and has a stent retriever diameter profile of 2 mm (3). The Trevo stent retriever, which was used in the first reported BEMP technique (1), has a minimum low profile of 3 mm, and a microcatheter can be used only up to 0.0175 in. (0.44 mm), whereas, the Tron stent retriever has a minimum profile of 2 mm, and a catheter can be used up to 0.0165 in. These differences provide the advantage that the Tron stent retriever can be deployed distally, and that less friction between the stent retriever and vessel wall is also expected to reduce hemorrhagic events (3). Furthermore, the Tron stent retriever is more useful for MCA M2 occlusion thrombectomy in the BEMP technique. Although the traditional combination technique using a Tron stent retriever with a 3MAX or 4MAX aspiration catheter may be effective in enhancing the first-pass effect (FPE) (7) in MCA M2 occlusion thrombectomy, we occasionally experience that a 4MAX aspiration catheter is often outsized for the target vessel, and that a 3MAX aspiration catheter for the target thrombus volume is insufficient when trying to engage these aspiration catheters proximal to the thrombus. In the BEMP technique, the aspiration catheter (3MAX or 4MAX aspiration catheter) can be determined after confirming the target vessel diameter (1, 2). Therefore, the BEMP technique using Tron stent retriever has potential as an effective and safe treatment for MeVO, including occlusion of the MCA M2. Here, we report the outcomes of the BEMP technique using Tron stent retriever for MCA M2 occlusion thrombectomy.

## Materials and Methods

### Study Design

All AIS patients admitted to our institute within 7 days from symptom onset or last-known-well date were prospectively registered in the National Cerebral and Cardiovascular Center (NCVC) Stroke Registry (8–13). Data for AIS patients with MCA M2 occlusion undergoing the BEMP technique were collected retrospectively from the NCVC Stroke Registry from March 2019 to June 2020. The decision to proceed with endovascular therapy (EVT), including the BEMP technique, was made at the discretion of the investigators in a nonrandomized fashion. This study was approved by the institutional review board of the NCVC (approval number: M23-073-4). The NCVC Stroke Registry is registered at ClinicalTrials.gov (NCT02251665).

### Tron Stent Retriever

**Figure 1:**
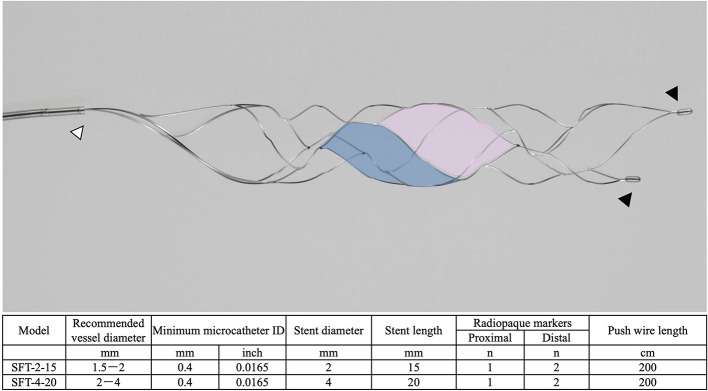
The details of the Tron stent retriever. Photograph of the Tron stent retriever, which is constructed of two types of cells. There are a radiopaque marker in the proximal end (white arrowhead) and two markers attached to the distal end (black arrowheads). The large cell (pink) captures the thrombus, and the small cell (blue) reduces stent extension at the flexion.

The Tron stent retriever is a self-expanding stent retriever made of a nickel–titanium alloy that consists of two types of cells with different shapes (3). The large cell captures the thrombus, and the small cell reduces stent extension at the flexion. When deploying and retracting from an elongated target vessel, the stent retriever stretches against the flexion force and passes through the lesser curvature side of an elongated target vessel. However, the unique cell structure of the Tron stent retriever allows the stent structure to stretch at the bend; therefore, this stent retriever is less likely to miss a blood clot. The details of the Tron stent retriever are illustrated in Figure 1.

### Interventional Protocol With the BEMP Technique Using the Tron Stent Retriever

**Figure 2:**
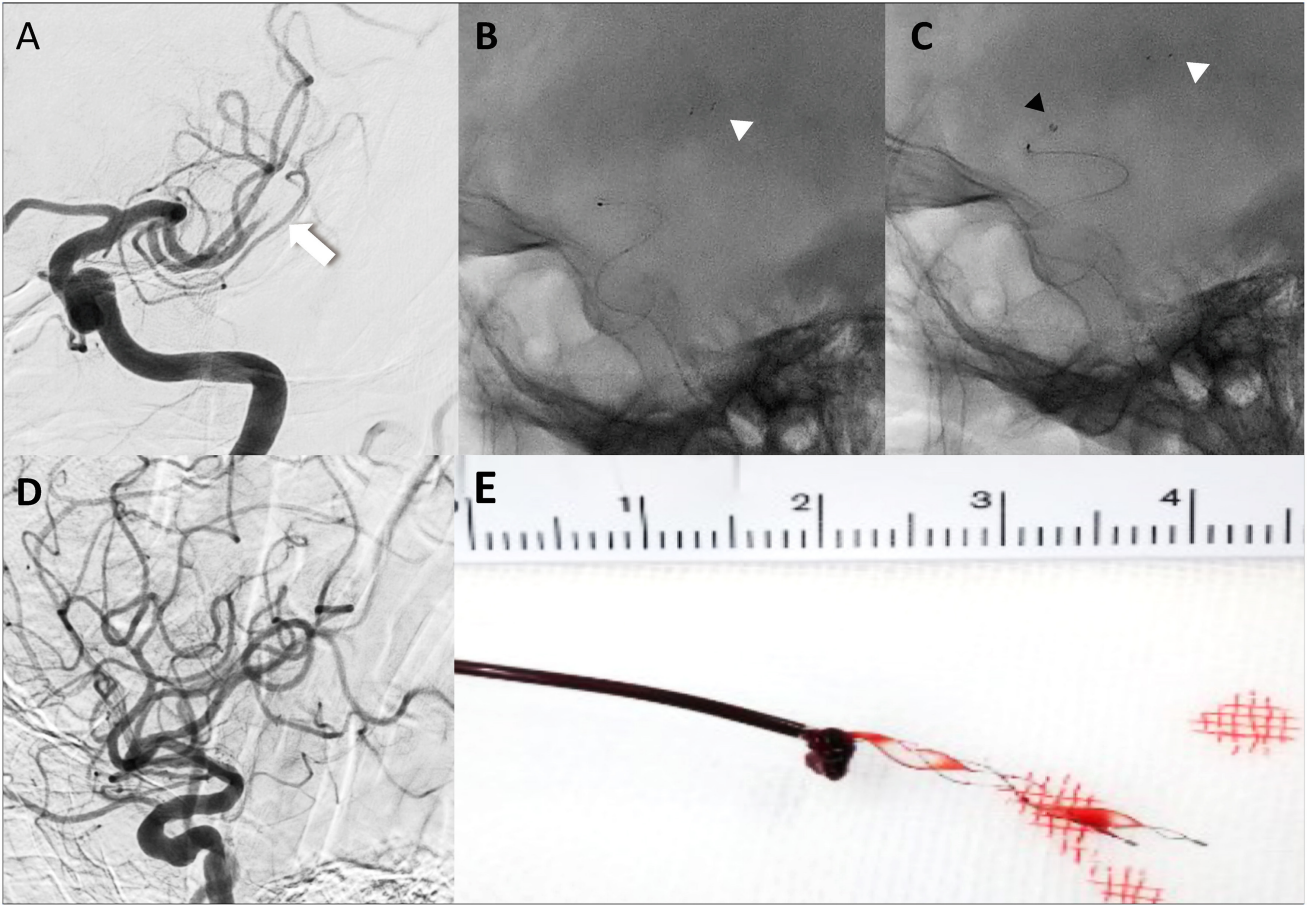
Illustrative cases of the blind exchange and mini-pinning technique using a Tron stent retriever to remove a clot. Illustration of blind exchange and mini-pinning using the Tron stent retriever in a dominant and proximal middle cerebral artery M2 occlusion. (white arrow: proximal end of the clot) **(A)** Lateral angiogram showing an M2 occlusion. **(B)** A 0.0165-inch microcatheter in place after deployment (white arrowhead: distal end of the retriever). **(C)** The 3MAX aspiration catheter is advanced into the face of the clot (black arrowhead) over the bare retriever delivery wire. **(D)** Final angiogram showing successful reperfusion. **(E)** Mini-pinning (2-mm Tron stent retriever and 3MAX aspiration catheter) with “corking” of the thrombus.

**Figure 3:**
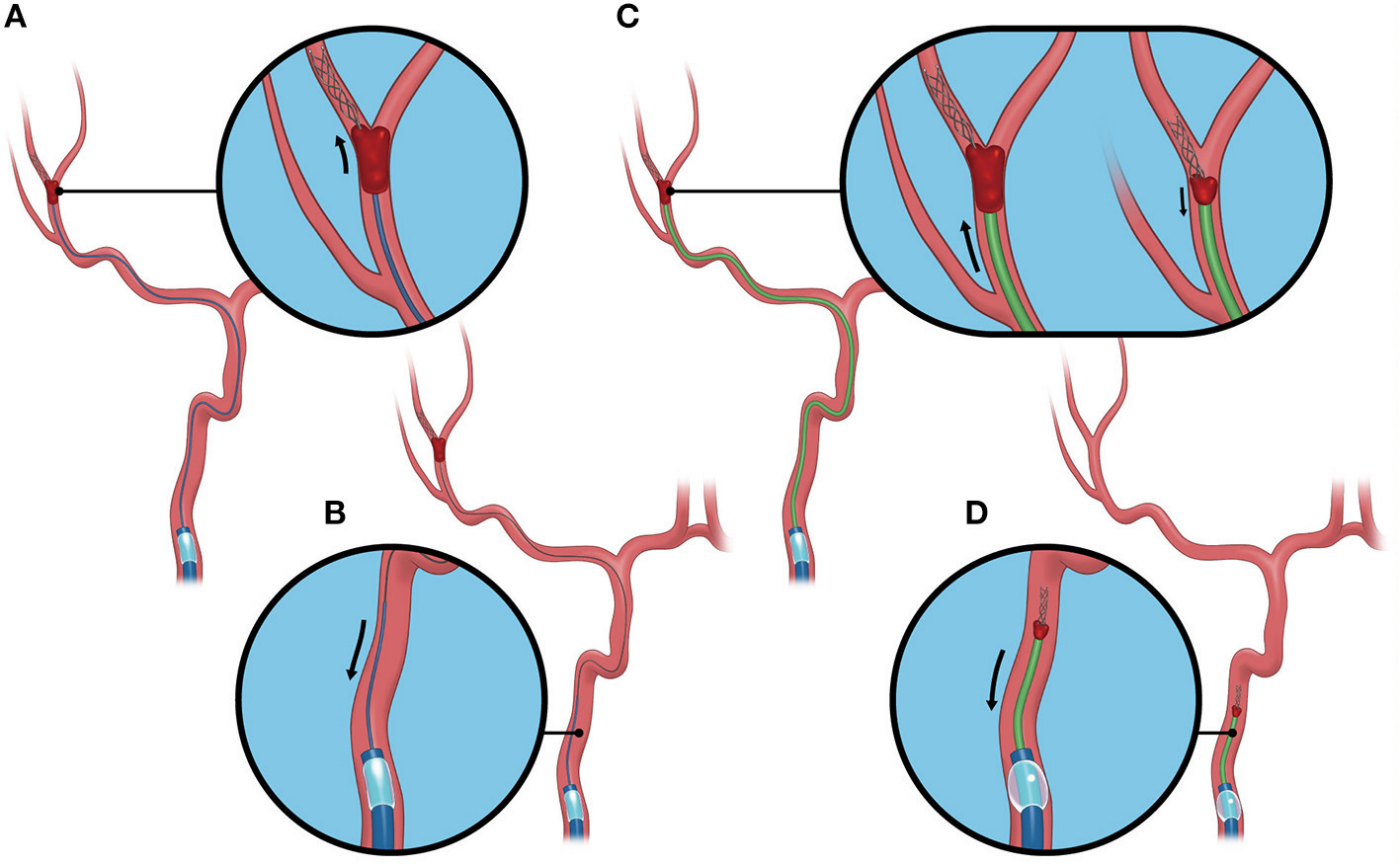
Blind exchange and mini-pinning using a Tron stent retriever to retrieve a clot. Illustrative example of the blind exchange and mini-pinning technique using a Tron stent retriever to treat a middle cerebral artery M2 occlusion. We routinely place a large-bore balloon guide catheter in the cervical internal carotid artery. **(A)** The biaxial system, including the microcatheter and microguidewire, is advanced as a unit, with the microcatheter and guidewire traversing the occluded segment. The Tron stent retriever is deployed *via* a 0.0165-inch microcatheter distal to the clot. **(B)** In the next step, the microcatheter is removed from the body. **(C)** A 3MAX or 4MAX aspiration catheter, as a distal aspiration catheter, is blindly navigated over the bare Tron delivery wire (“blind exchange”) until the aspiration catheter reaches the clot. (**C**, left) The Tron stent retriever is pulled into the distal aspiration catheter like placing a bottle cork, and the pinned clot with the stent retriever and the distal aspiration catheters then forms a single unit. (**C**, right). **(D)** The Tron stent retriever and aspiration catheter are subsequently retracted as a unit to retrieve the clot.

All procedures in the BEMP technique using the Tron stent retriever were performed by four experienced neurointerventionalists (TY, KTa, JK, and HY) as a front-line thrombectomy with local anesthesia or conscious sedation. The BEMP technique was performed through a right femoral artery approach, and the occluded-side cervical internal carotid artery (ICA) was catheterized using a balloon guide catheter (BGC). The proximal BGC used in all procedures was a 9-Fr Optimo BGC (Tokai Medical Products Inc., Aichi, Japan). The BEMP technique using the Tron stent retriever is performed initially by advancing a 0.0165-inch microcatheter (Excelsior SL-10 microcatheter; Target Therapeutics/Stryker, Fremont, CA, USA) distal to the occlusion site. Next, a 2 × 15-mm or 4 × 20-mm Tron stent retriever (JIMRO; Terumo, Takasaki, Gunma, Japan) is deployed *via* a 0.0165-inch microcatheter, followed by microcatheter removal and immediate blind navigation of a low-profile distal aspiration catheter (DAC), namely, a 3MAX or 4MAX aspiration catheter (Penumbra, Alameda, CA, USA), over the bare Tron delivery wire (“blind exchange”) until the DAC reaches the clot. The aspiration catheter is then aspirated using a commercially available aspiration pump. The Tron stent retriever is pulled into the DAC like placing a bottle cork, and the pinned clot with the stent retriever and the DAC then forms a single unit. The Tron stent retriever and DAC are subsequently retracted as a unit to retrieve the clot (1). Figure 2 shows the BEMP technique performed as the primary maneuver. Figure 3 illustrates an example of the BEMP technique using the Tron stent retriever for MCA M2 occlusion.

### Clinical Data Collection and Definitions

The baseline clinical characteristics for the following variables were collected: sex, age, prestroke modified Rankin Scale (mRS) score, medical history (hypertension, dyslipidemia, diabetes mellitus, current smoking, ischemic stroke, and atrial fibrillation), systolic blood pressure at admission, baseline National Institutes of Health Stroke Scale (NIHSS) score, baseline Alberta Stroke Program Early Computed Tomographic Score (ASPECTS) on diffusion-weighted imaging (DWI) or computed tomography (CT), baseline ischemic core volume, time logistics (onset-to-groin puncture time and groin puncture-to-revascularization time), treatment profile (intravenous thrombolysis), type of presentation by vessel occlusion (isolated M2 occlusion, tandem occlusion, or multi-vessel M2 occlusion), M2 occlusion site (proximal, distal), M2 type (dominant, co-dominant, or non-dominant), M2 division (superior, inferior), and stroke causative mechanism. Baseline ischemic core volume was calculated according to an apparent diffusion coefficient of <620 × 10^−6^ mm^2^/s (14) on DWI or a relative reduction in cerebral blood flow of <30% (15) on CT perfusion using an automated image postprocessing system (RAPID, version 4.9.2.2; iSchemaView, Menlo Park, CA, USA). Hypoperfused tissue, which represents the penumbral tissue, was estimated in accordance with previously validated thresholds as time-to-maximum (Tmax) >6 s (16, 17). Occlusion sites were determined using digital subtraction angiography. The stroke causative mechanism was determined according to the Trial of ORG 10172 in Acute Stroke Treatment criteria by board-certified stroke neurologists.

### M2 Definition

The MCA M2 segment was defined as the vessels from the main MCA bifurcation/trifurcation to the circular sulcus of the insula (18, 19). M2 occlusions in which the occluded MCA bifurcated after the horizontal segment were considered proximal occlusions. Proximal M2 was defined as the horizontal M2 segment from the main MCA bifurcation/trifurcation within 1 cm from the MCA bifurcation and distal M2 as the Sylvian M2 segment, starting 1 cm from the bifurcation/trifurcation to the circular sulcus of the insula (19–21). M2 caliber dominance was considered present if the M2 branch had a larger diameter than the other branches on digital subtraction angiography or if the perfusion defect associated with the occluded M2 branch was larger than 50% of the MCA territory. Only when the diameters of both the inferior and superior branches were equal and the associated perfusion defect was <50% of the MCA territory, were the branches considered co-dominant (22). M2 caliber dominance can be complicated to determine with initial angiograms, such as when the vessel is small but secondary to occlusion. Therefore, we also referred to hypoperfused tissue volume (Tmax: >6 s) to determine M2 caliber dominance. Angiograms were evaluated for the presence of M2 occlusion, and occlusions were classified according to their clinical scenario: isolated M2 occlusion, tandem occlusion, or multi-vessel M2 occlusion. Tandem occlusion was defined as an M2 occlusion with a concomitant larger vessel/proximal occlusion. Multi-vessel M2 occlusion was defined as ipsilateral occlusions of the M2 superior trunk of the MCA and M2 inferior trunk of the MCA.

### Outcomes

The procedural outcomes were defined as a first-pass expanded thrombolysis in cerebral infarction (eTICI) score of 2c/3 and 2b/2c/3 for the targeted M2 (23). Safety outcomes were defined as the presence of any parenchymal hematoma (PH) [hemorrhagic infarction according to the European Cooperative Acute Stroke Study (ECASS); PH type 1 or 2], any intracranial hemorrhage (ICH), symptomatic ICH (sICH), which was defined according to the ECASS II criteria (any ICH with a ≥4-point increase in the NIHSS score from baseline) (24), subarachnoid hemorrhage (SAH) (diffuse or focal within the territory of the artery treated for distal occlusion), arterial spasm (defined as any degree of spasm in treated vessels), emboli to the same or different territory after thrombectomy of a proximal occlusion, active extravasation, and mortality at 90 days. Efficacy outcome was defined as the rate of good outcome (mRS score of 0–2 at 90 days).

### Statistical Analysis

Continuous variables are reported as median [interquartile range (IQR)], and categorical variables are reported as proportions. Analyses were performed using Stata 15.1 (StataCorp, College Station, TX, USA).

## Results

**Figure 4:**
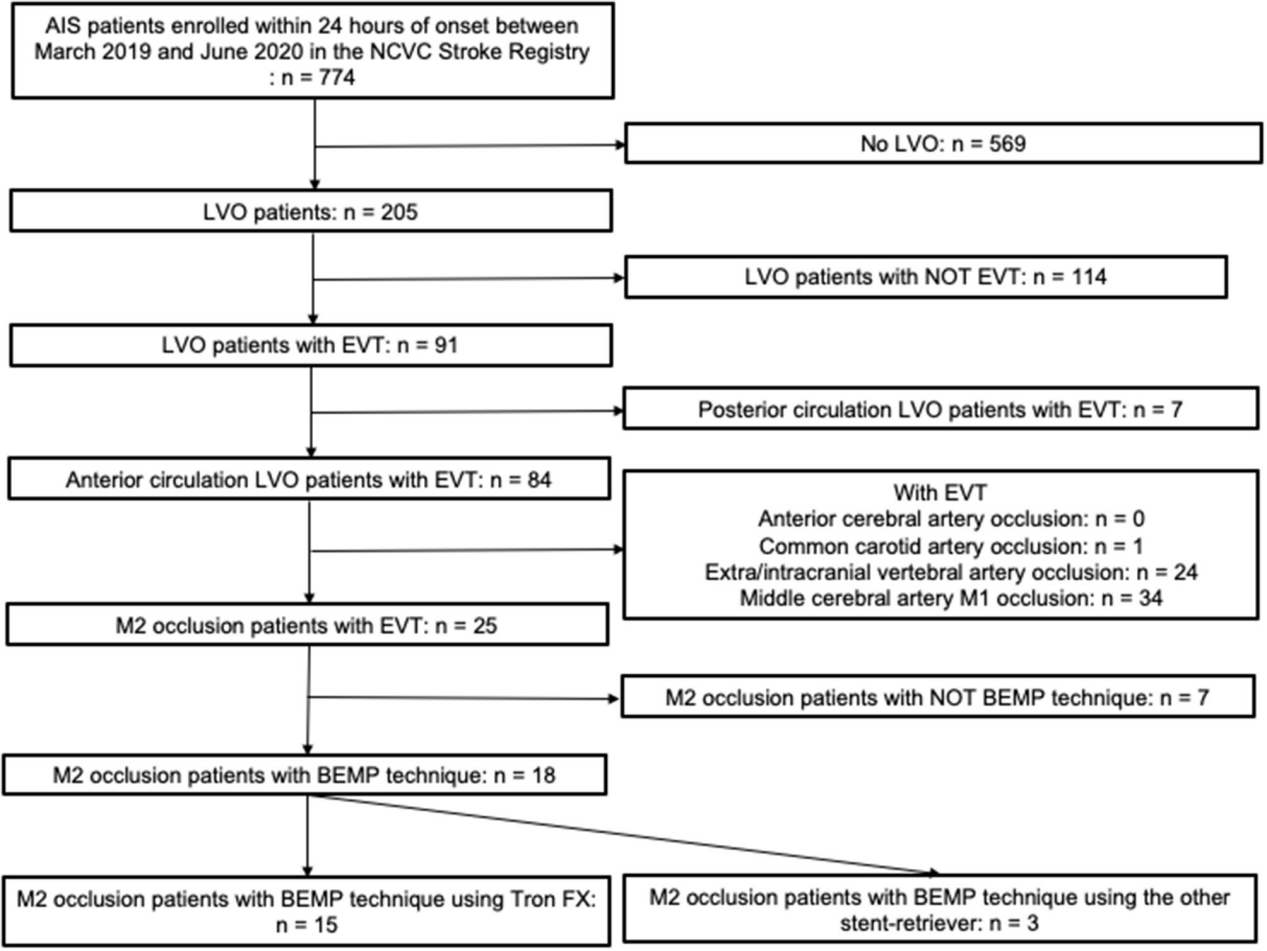
Study flowchart. ACA, anterior cerebral artery; BA, basilar artery; BEMP, blind exchange with mini-pinning; BGC, balloon guide catheter; CCA, common carotid artery; EVT, endovascular therapy; ICA, internal carotid artery; MCA, middle cerebral artery; NCVC, National Cerebral and Cardiovascular Center; PCA, posterior cerebral artery; VA, vertebral artery.

The study flowchart is provided in Figure 4. A total of 774 patients had a diagnosis of AIS within 24 h of onset between March 2019 and June 2020 in the NCVC Stroke Registry, and of these, 62 patients had MCA M2 occlusion, including 25 patients who underwent EVT. Of the 25 M2 occlusion patients, 18 MCA M2 occlusion patients were treated with the BEMP technique. Of the seven patients who did not undergo the BEMP technique, two patients were enrolled in another device clinical trial, and in one patient, we were unable to advance the BGC into the ICA; this patient underwent a simple stent retrieval technique. The remaining four patients underwent another EVT technique (Aspiration-Retriever Technique for Stroke technique (25) using the EmboTrap II device [Neuravi/Cerenovus, Miami, FL, USA] or a Trevo stent retriever and a large-bore aspiration catheter) at the investigator's discretion. The BEMP technique using a Trevo stent retriever was performed in three patients before the use of the Tron stent retriever was approved.

Six patients (40%) were female; the median (IQR) age was 80 (75–83) years; the median NIHSS score was 18 (11–25); baseline ASPECTS on DWI or CT: 7 (6–10); onset-to-groin puncture time: 141 (101–170) min. The median groin puncture-to-revascularization time was 55 (45–75) min, of which the median groin puncture-to-revascularization time in isolated M2 occlusion was 35 (27–41) min, and that for tandem occlusion or multi-vessel M2 occlusion was 82 (58–112) min. Intravenous alteplase was used in 9 (60%) patients, and a BGC was used in 15 (100%) patients; eight patients had isolated M2 occlusions. Tandem occlusion included the M2 plus the intracranial ICA (*n* = 3) or MCA M1 (*n* = 1). Three patients had multi-vessel M2 occlusion. At the M2 occlusion site, 11/18 (61%) vessels had proximal M2 occlusion, and 7/18 vessels had distal M2 occlusion. Dominance of either superior or inferior division was observed in 12/15 (80%) patients, and co-dominance of M2 branches was observed in 3 (20%) patients with M2 occlusion. Dominance of a branch was more commonly seen in the inferior division (7/10). A first-pass eTICI score of 2c/3 was achieved in 7/15 (47%) vessels, and a first-pass eTICI score of 2b/2c/3 was achieved in 9/15 (60%) vessels. A final eTICI score of 2c/3 was obtained in nine patients (60%), and a final eTICI score of 2b/2c/3 was obtained in 14/15 patients (93%). The median (IQR) number of passes was 1 (1–3).

Concerning the safety and efficacy outcomes, PH, SAH, arterial spasm, and emboli to the same territory after thrombectomy of a proximal occlusion were seen in one patient each, respectively. ICH was seen in 6 patients (40%); no sICH, active extravasation, or emboli to the different territory after thrombectomy of a proximal occlusion were observed. The rate of good outcome was 47% (7/15), and 1 patient died with a history of severe heart failure. The patients' baseline characteristics and outcomes are summarized in Table 1, and the cohort clinical outcomes and the outcomes in the M2 occlusion patients undergoing BEMP using the Tron stent retriever and other stent retrievers (Trevo stent retriever) are summarized in Table 2.

**Table 1:** Baseline characteristics and outcomes.

	**BEMP technique**
	***N* = 15 patients (18 vessels)**
**Baseline characteristics**	
Female	6/15 (40)
Age, years	80 (75–83)
Prestroke mRS score	0 (0–2)
Hypertension	12/15 (80)
Dyslipidemia	7/15 (47)
Diabetes mellitus	4/15 (27)
Current smoking	4/15 (27)
Ischemic stroke	2/15 (13)
Atrial fibrillation	10/15 (67)
Systolic blood pressure at admission, mmHg	152 (143–169)
Baseline NIHSS score	18 (11–25)
Baseline ASPECTS on DWI or CT	7 (6–10)
Baseline ischemic core volume, mL	31 (8–67)
Intravenous alteplase	9/15 (60)
**Time logistics**	
Onset-to-groin puncture time, min	141 (101–170)
Groin puncture-to-revascularization time, min	55 (45–75)
Groin puncture-to-revascularization time in the isolated M2 occlusion, min (*n* = 8)	35 (27–41)
**Type of presentation (by vessel)**	
Isolated M2 occlusion	8/15 (53)
Tandem occlusion	4/15 (27)
Multi-vessel M2 occlusion	3/15 (20)
**M2 occlusion site (*****N*** **=** **18)**	
Proximal M2	11/18 (61)
Distal M2	7/18 (39)
**M2 type (*****N*** **=** **18)**	
Dominant M2	10/18 (56)
Co-dominant M2	3/18 (17)
Non-dominant M2	5/18 (28)
**M2 division (*****N*** **=** **18)**	
Superior M2	8/18 (44)
Inferior M2	10/18 (56)
**Aspiration catheter (*****N*** **=** **18)**	
3MAX	9/18 (50)
4MAX	9/18 (50)
**Stroke causative mechanism**	
Large artery atherosclerosis	2/15 (13)
Cardioembolic	9/15 (60)
Undetermined	4/15 (27)
**Outcomes**	
**Procedural outcomes**	
First-pass eTICI score 2c/3	7/15 (47)
First-pass eTICI score 2b/2c/3	9/15 (60)
Final eTICI score 2c/3	9/15 (60)
Final eTICI score 2b/2c/3	14/15 (93)
The number of passes	1 (1–2)
**Safety outcomes**	
Parenchymal hematoma	1/15 (7)
Any ICH	6/15 (40)
Symptomatic ICH	0
Subarachnoid hemorrhage	1/15 (7)
Arterial spasm	1/15 (7)
Emboli to the same territory after thrombectomy of a proximal occlusion	1/15 (7)
Emboli to the different territory after thrombectomy of a proximal occlusion	0
Active extravasation	0
Mortality at 90 days	1/15 (7)
**Efficacy outcomes**	
mRS score of 0–2 at 90 days	7/15 (47)

*Data are presented as number (%) or median (interquartile range)*.

*ASPECTS, Alberta Stroke Program Early Computed Tomographic Score; BEMP, blind exchange with mini-pinning; CT, computed tomography; DWI, diffusion-weighted imaging; eTICI, expanded Thrombolysis in Cerebral Infarction; ICH, intracranial hemorrhage; mRS, modified Rankin Scale; NIHSS, National Institutes of Health Stroke Scale*.

**Table 2:** The clinical characteristics and outcomes in MCA M2 occlusion patients undergoing the BEMP technique using the Tron vs. other stent retrievers.

**Stent retriever**	**Profile**	**Patient no**.	**Vessel no**.	**Age, years**	**Core volume, mL**	**Tmax >6 s volume, mL**	**Site**	**Proximal/distal**	**Dominant**	**Divisions**	**DAC**	**First-pass eTICI score 2c/3**	**First-pass eTICI score 2b/2c/3**	**Final eTICI score**	**Any ICH**	**PH**	**3M-mRS**
Tron	2 mm	1	1	79	0	62	L	Proximal	Dominant	Inferior	4MAX	N	N	2b	N	N	4
Tron	2 mm	2	2	78	8	NA	R	Distal	Non-dominant	Superior	3MAX	Y	Y	2b	N	N	1
Tron	4 mm	3	3	75	85	NA	R	Proximal	Dominant	Inferior	4MAX	N	N	2a	Y	Y	5
Tron	4 mm	4	4	83	17	NA	R	Proximal	Dominant	Inferior	4MAX	N	Y	3	N	N	4
Tron	2 mm	4	5	–	–	–	R	Proximal	Non-dominant	Superior	3MAX	N	Y	–	–	–	–
Tron	4 mm	5	6	81	0	NA	L	Distal	Dominant	Inferior	4MAX	Y	Y	2c	N	N	3
Tron	2 mm	6	7	81	0	NA	L	Distal	Non-dominant	Superior	3MAX	N	N	3	Y	N	1
Tron	2 mm	6	8	–	–	–	L	Proximal	Dominant	Inferior	3MAX	N	N	–	–	–	–
Tron	2 mm	7	9	80	0	NA	L	Distal	Co-dominant	Superior	3MAX	Y	Y	2c	N	N	0
Tron	4 mm	8	10	81	64	185	L	Proximal	Dominant	Superior	4MAX	N	N	3	Y	N	4
Tron	4 mm	9	11	83	67	94	L	Proximal	Dominant	Inferior	4MAX	Y	Y	2c	Y	N	6
Tron	2 mm	10	12	91	29	NA	R	Distal	Dominant	inferior	4MAX	N	Y	2b	Y	N	5
Tron	2 mm	11	13	71	8	61	L	Distal	Co-dominant	Inferior	3MAX	N	N	2b	N	N	1
Tron	2 mm	12	14	52	32	47	R	Proximal	Co-dominant	Inferior	4MAX	Y	Y	2b	N	N	0
Tron	2 mm	13	15	75	5	71	L	Distal	Dominant	Superior	4MAX	Y	Y	2c	Y	N	1
Tron	4 mm	14	16	71	32	NA	L	Proximal	Non-dominant	Superior	3MAX	Y	Y	2c	N	N	0
Tron	2 mm	15	17	85	74	NA	L	Distal	Dominant	Superior	3MAX	N	N	3	N	N	5
Tron	2 mm	15	18	–	–	–	L	Proximal	Non-dominant	Inferior	3MAX	N	N	–	–	–	–
	The other stent retriever																
Trevo	4 mm	–	–	–	12	114	L	Proximal	Dominant	Inferior	4MAX	N	N	0	Y	N	4
Trevo	3 mm	–	–	–	8	58	R	Proximal	Co-dominant	Superior	3MAX	N	N	2b	Y	N	3
Trevo	3 mm	–	–	–	21	74	L	Proximal	Dominant	Inferior	3MAX	N	N	2b	Y	N	3

*BEMP, blind exchange with mini-pinning; DAC, distal aspiration catheter; eTICI, expanded Thrombolysis in Cerebral Infarction; ICH, intracranial hemorrhage; MCA, middle cerebral artery; mRS, modified Rankin Scale; NA, not applicable; PH, parenchymal hematoma; Tmax, time-to-maximum*.

In seven patients with MCA M2 occlusion who did not undergo the BEMP technique, the rate of a first-pass eTICI score of 2c/3 was 29% (2/7), and the rate of a first-pass eTICI score of 2b/2c/3 was 43% (3/7). ICH was seen in four patients (57%); no sICH or active extravasation was observed. The rate of good outcome was 43% (3/7).

## Discussion

We describe the BEMP technique using the Tron stent retriever, which appears to be helpful and safe in optimizing the procedural performance in the treatment of MCA M2 occlusion. In the traditional combination technique, we sometimes experience that a 4MAX aspiration catheter (1.42-mm distal outer diameter) is outsized for the target vessel, and a 3MAX aspiration catheter (1.27-mm distal outer diameter) is insufficient for the target thrombus volumes. Therefore, before navigating these catheters in a coaxial manner, it is necessary to thoroughly judge what types of aspiration catheters are required in advance concerning the diameter and sites of the occluded MCA M2 vessels. The blind exchange technique was derived from the need to circumvent this limitation. Furthermore, the stiffness of the retriever delivery wire, distal anchoring by the deployed Tron stent retriever, which has less friction on M2 vessels, and coverage of the wire by the BGC eliminate the risk of pushing/losing the delivery wire in the patient. The subsequent straightening of the vessels that can be achieved by gently pulling the retriever wire greatly facilitates overcoming the barriers to M2 navigation of DACs. Pérez-García et al. reported that the BEMP technique using 3 × 15-mm, 3 × 20-mm, or 3.5 × 28-mm stent retrievers led to higher the first-pass eTICI 2c/3 recanalization rates (57.1%) and a lower incidence of sICH (1.9%) than that using mini-stent retrievers alone in thrombectomy for intracranial MeVOs (2). In our BEMP technique using 2 × 15-mm or 4 × 20-mm Tron stent retrievers, the first-pass eTICI 2c/3 recanalization was observed in 47% of the M2 vessels, and no sICH was seen for MCA M2 occlusion. These results may indicate clinical benefit in the BEMP technique using Tron stent retrievers for MCA M2 occlusion. Additionally, the minimum sizes of low-profile stent retrievers reported in the BEMP technique were in the 3-mm range (Aperio: 3.5 mm and Catch Mini: 3 mm). Few reports of the BEMP technique used tiny, 2-mm stent retrievers. By combining a stent retriever with a 2-mm profile and the effective BEMP technique, it may be possible to more effectively treat MeVO, such as M2 distal and M3 occlusions, which have been somewhat challenging.

The concept of the BEMP technique using the Tron stent retriever for MCA M2 occlusion is to deploy the retriever and advance the DAC until it reaches the clot. Relatively similar techniques for proximal occlusions have been described, mostly consisting of single-arm studies (25–31) and some comparative studies (26, 30–32) showing that the combined use of contact aspiration and the stent retriever technique may be superior to either retriever or aspiration thrombectomy alone. However, there are few reports of techniques specifically analyzing MCA M2 occlusions. The critical aim of the BEMP technique using the Tron stent retriever is to push the DAC slightly past the proximal end of the clot in order to “cork” (partially insert) the thrombus into the catheter and to pull the Tron stent retriever a little into the DAC to “plug” the DAC with the thrombus. This increases the retrieval force by combining the retriever's traction with aspiration, which may explain the relatively higher rates of the first-pass eTICI 2b/2c/3 and the trend toward increased the first-pass eTICI 2c/3 in our results. Moreover, the friction for retrieval is attenuated because less retriever is exposed, consequently decreasing stretching and torsion in tortuous and small distal vessels (32).

It has been reported that the outer diameters of MCA M2 and M3 segments are 1.4–2.3 and 0.8–1.5 mm, respectively (33). The diameters of previous reported mini-stent retrievers are 3–3.5 mm (1, 2, 6), which is considered slightly large for MCA M2 or M3 segments (34). Since the Tron stent retriever has a 2-mm profile, it can be safely deployed even in non-dominant distal M2 occlusions with a small diameter. Finally, by adding the distal support in the clot's vicinity, the force vector is optimized as it approaches the angle of movement. The frequency of hemorrhagic complications was relatively small, in our study, and only one PH, which was caused by recanalization, could be directly attributed to the BEMP technique for MCA M2 occlusion. Although the number was as small as three, any ICH frequency was almost the same as that of the BEMP technique using the Tron stent retriever compared with other stent retrievers. Additionally, although the effect of clot composition on the performance of the different techniques is not well-defined, this appears to play an integral role in procedural performance (31, 32).

One of the present technique's strengths is that an optimal DAC (4MAX or 3MAX aspiration catheter) can be determined after deploying a stent retriever, confirming intermediate flow restoration, and determining the target vessel diameter if the combined use of contact aspiration and the stent retriever technique is considered effective (1, 26). By capturing part of the Tron stent retriever by the low-profile DAC (mini-pinning), there is less contact surface between the stent retriever and the arterial wall, reducing the radial and tractional force exerted by the stent retriever on medium-sized branches and reducing the risk of vessel laceration in MCA M2 thrombectomy. However, the following should be noted when performing the BEMP technique with the Tron stent retriever: if the inside of the aspiration catheter is not coated, such as with the SOFIA (MicroVention, Tustin, CA, USA), the inside of the aspiration catheter may be damaged when blindly guiding the aspiration catheter to the proximal thrombus. Moreover, several pitfalls with the BEMP technique should be noted. When the ICA bifurcation was strongly angled or when the BGC was placed in the common carotid artery owing to difficulty in access, the bare Tron delivery wire flexed more strongly, and the DAC entered the external carotid artery. The key to addressing this pitfall is to follow the bare Tron delivery wire by advancing the DAC while removing the deflection by pulling the bare Tron delivery wire slightly, without forcing the DAC to advance. In our study, the BEMP technique with the Tron stent retriever caused this pitfall in 3/15 patients, but the above method solved this problem in all three patients.

This study has several limitations, and the first limitation was the small sample size. Second, the BEMP technique using the Tron stent retriever cannot be compared with the BEMP technique using other devices or another technique, including a simple stent retrieval technique, contact aspiration, or the combined use of contact aspiration the stent retriever technique. Third, because no formal protocol for device selection was followed, unmeasured variables could have introduced bias. Finally, the costs related to the use of an additional device must be carefully considered. However, our results are encouraging, and further studies are justified.

In conclusion, the Tron stent retriever was safely and effectively used in the BEMP technique for acute MCA M2 occlusion, which was originally devised using the Trevo 3 × 20-mm retriever (1). The features of the Tron stent retriever allow it to be combined with 0.0165-inch microcatheters, and this device may be useful for treating MeVO. Our results might indicate an extended indication and emerging thrombectomy technique in AIS to M2 and M3 MeVO using the Tron stent retriever.

## Data Availability Statement

The original contributions generated for this study are included in the article/supplementary material, further inquiries can be directed to the corresponding author/s.

## Ethics Statement

The studies involving human participants were reviewed and approved by this study was approved by the institutional review board of the NCVC (approval number: M23-073-4). The NCVC Stroke Registry is registered at ClinicalTrials.gov (NCT02251665). The patients/participants provided their written informed consent to participate in this study. Written informed consent was obtained from the individual(s) for the publication of any potentially identifiable images or data included in this article.

## Author Contributions

TY, KTa, JK, and HY: study conception. TY, KTa, and JK: acquisition of data. TY: analysis and interpretation of the data and drafting the manuscript. TY, KTa, JK, TS, KTo, and MK: editing the manuscript for intellectual content. All authors revising the manuscript critically for important intellectual content, final approval of the version to be published, and agreement to be accountable for all aspects of the work and ensuring that questions related to the accuracy or integrity of any part of the work are appropriately investigated and resolved.

## Conflict of Interest

The authors declare that the research was conducted in the absence of any commercial or financial relationships that could be construed as a potential conflict of interest.
